# A novel role for drebrin in regulating progranulin bioactivity in bladder cancer

**DOI:** 10.18632/oncotarget.3424

**Published:** 2015-03-12

**Authors:** Shi-Qiong Xu, Simone Buraschi, Alaide Morcavallo, Marco Genua, Tomoaki Shirao, Stephen C. Peiper, Leonard G. Gomella, Ruth Birbe, Antonino Belfiore, Renato V. Iozzo, Andrea Morrione

**Affiliations:** ^1^ Department of Urology and Biology of Prostate Cancer Program, Thomas Jefferson University, Philadelphia, PA, USA; ^2^ Department of Pathology, Anatomy and Cell Biology and Cancer Cell Biology and Signaling Program, Kimmel Cancer Center, Thomas Jefferson University, Philadelphia, PA, USA; ^3^ Department of Health and Endocrinology, University Magna Graecia of Catanzaro, Catanzaro, Italy; ^4^ Department of Neurobiology and Behavior, Gunma University School of Medicine, Showamachi, Maebashi, Japan

**Keywords:** Progranulin, drebrin, bladder cancer, migration, invasion and anchorage-independent growth

## Abstract

We recently established a critical role for the growth factor progranulin in bladder cancer insofar as progranulin promotes urothelial cancer cell motility and contributes, as an autocrine growth factor, to the transformed phenotype by modulating invasion and anchorage-independent growth. In addition, progranulin expression is upregulated in invasive bladder cancer tissues compared to normal controls. However, the molecular mechanisms of progranulin action in bladder cancer have not been fully elucidated. In this study, we searched for novel progranulin-interacting proteins using pull-down assays with recombinant progranulin and proteomics. We discovered that drebrin, an F-actin binding protein, bound progranulin in urothelial cancer cells. We characterized drebrin function in urothelial cancer cell lines and showed that drebrin is critical for progranulin-dependent activation of the Akt and MAPK pathways and modulates motility, invasion and anchorage-independent growth. In addition, drebrin regulates tumor formation *in vivo* and its expression is upregulated in bladder cancer tissues compared to normal tissue controls. Our data are translationally relevant as indicate that drebrin exerts an essential functional role in the regulation of progranulin action and may constitute a novel target for therapeutic intervention in bladder tumors. In addition, drebrin may serve as novel biomarker for bladder cancer.

## INTRODUCTION

Bladder cancer (BC) is one of the most common cancers in the United States with 56,390 estimated new cases and 11,170 estimated deaths in 2014 [1]. Bladder tumors show diverse histopathological patterns and clinical behaviors [2] making this a serious hindrance in the management of bladder tumors. The clinical stage of BC is dependent on the depth of tumor invasion [3] and, although the prognosis for low-grade tumors is generally good, 10%–15% of these patients will later develop invasive disease. For invasive tumors instead, prognosis is much less favorable, with only 50% survival at 5 years. Invasive tumors frequently progress to life-threatening metastases, which are associated with a 5 year survival rate of ~6% [2]. Thus, understanding the mechanisms that regulate bladder tumor invasion and progression toward metastasis is critical to predict and treat this devastating condition in bladder cancer patients.

The growth factor progranulin, also known as proepithelin, PCDGF, granulin-epithelin precursor or acrogranin, is a secreted glycoprotein which plays an important role in cell proliferation, wound healing and transformation in several cancer model systems [4–6]. In addition, progranulin regulates inflammation and neurodegeneration [7] and low levels of circulating progranulin are associated with fronto-temporal dementia (FTD). Progranulin interacts with the protein core of the heparan sulfate proteoglycan perlecan and it has been suggested that this interaction leads to a pro-angiogenic and proinvasive phenotype [8]. We have discovered that progranulin plays a critical role in bladder cancer by promoting urothelial cancer cell motility [9], and demonstrated that progranulin contributes as an autocrine growth factor to the transforming phenotype by regulating invasion and anchorage-independent growth [10, 11]. In addition, progranulin is overepressed in invasive bladder cancer tissues vis-à-vis non-neoplastic tissues and it is detectable in the urine [11]. Thus, progranulin may be critical for the transition to the invasive phenotype in bladder cancer and may serve as a novel biomarker for bladder cancer.

Despite the increasing interest in progranulin biology, progranulin's mode of action is still poorly characterized. Furthermore, very few proteins that mediate the early stages of progranulin signaling from the plasma membrane have been so far characterized.

Previous studies have identified proteins of ~130 kDa as putative progranulin receptors [12, 13] and more recently, sortilin has been identified as a novel progranulin-interacting protein using membrane binding with alkaline phosphatase-labeled progranulin. Sortilin is a single-pass Type I transmembrane protein of the Vps10 family that is localized to the cell surface, secretory, and endocytic compartments of eukaryotic cells [14]. However, sortilin acts as negative regulator of progranulin levels and signaling by targeting progranulin for lysosomal degradation [14].

Progranulin has been shown to bind TNF receptors 1 and 2 and decrease TNF-dependent activation of MAPK (ERKs, p38 and JNK) by disturbing the TNF/TNFR interaction [15]. These results therefore do not support a role of TNFRs as *bona fide* progranulin signaling interacting partner. In addition, more recent papers have challenged the physical and functional interaction between progranulin and TNFRs [16], suggesting that additional experiments are necessary to clarify these contradictory results. In addition, in the presence of CpG-ONDs progranulin proteolytic fragments are soluble cofactors for Toll-like receptor 9 (TLR9) and contribute to innate immunity [17]. Furthermore, Tropomyosin 3 has been more recently reported as a novel progranulin-interacting protein in hepatocellular carcinoma cells [18], but the biological significance of this interaction has not been yet fully characterized.

In search for novel progranulin interacting proteins we performed pull-down assays with recombinant progranulin and protein extracts of 5637 bladder cancer cells. Proteomic analysis identified the F-actin-binding protein drebrin (developmentally regulated brain protein) [19, 20] as a novel progranulin-binding partner. We have characterized the biological significance of this interaction in invasive bladder cancer cells and showed that drebrin is critical for the regulation of progranulin-induced cell motility and invasion by mediating progranulin-induced F-acting remodeling. Furthermore, drebrin is essential for progranulin-induced activation of the Akt and MAPK signaling pathways. Significantly, drebrin depletion in tumorigenic urothelial cancer cells inhibits motility, anchorage-independent growth and tumor formation *in vivo*. In addition, drebrin is upregulated in high grade bladder cancer tissues compared to lower grade and normal tissue controls.

Collectively, we have identified drebrin as a novel progranulin-interacting protein and presented evidences that support a critical role for drebrin in regulating progranulin-dependent signaling and biological responses in bladder cancer.

## RESULTS

### Progranulin interacts with drebrin

**Figure 1:**
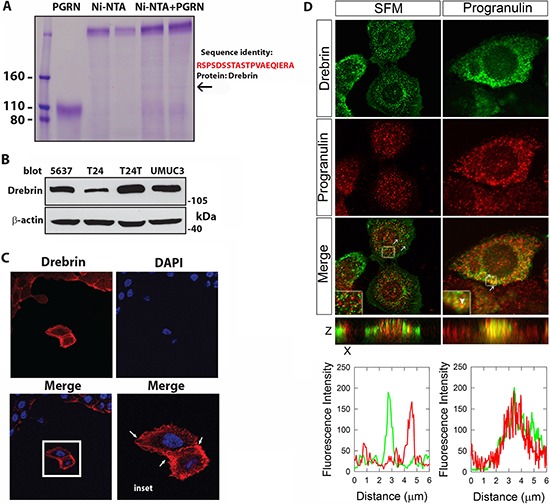
Progranulin interacts with the F-acting-binding protein drebrin **(A)** Proteomic identification of drebrin from 5637 cell extract pulled-down with recombinant His-tagged progranulin. Samples were separated by SDS-PAGE, visualized with Coomassie blue staining and processed for mass spectrometry. Bands isolated from gel and analyzed are identified by the arrow. Drebrin identity was confirmed on a local Mascot server against the Swissprot database. **(B)** Drebrin immunodetection from lysates of various urothelial-carcinoma cell lines (Upper panel). B-actin was used as a loading control (lower panel). The blot is representative of two independent experiments. **(C)** Representative images of immunofluorescence analysis of drebrin expression in 5637 cells. Arrows show drebrin localization at the membrane. DAPI staining was used to label cell nuclei. **(D)** Drebrin colocalizes with progranulin after 5 min of stimulation as determined by confocal microscopy. Notice the distinct co-localization of drebrin and progranulin in the Z stacks (yellow staining, bottom right panel). The line-scanned profiles at the bottom of the fluorescence images show the distribution of the fluorescence signals of each channel between the white arrows in the corresponding confocal images. Pictures are representative of at least 10 independent fields from three independent experiments. An average of 300 cells was examined for each condition.

In a search for novel progranulin interacting proteins we performed pull-down assays with recombinant progranulin and protein extracts of 5637 bladder cancer cells. Samples were separated by SDS-PAGE, visualized with Coomassie blue staining and processed for mass spectrometry. Proteomic analysis of selected bands (Figure 1A, arrow) identified the neuronal F-actin-binding protein drebrin (developmentally regulated brain protein) [19, 20] as a novel progranulin-interacting protein (Figure 1A, arrow). Next, we confirmed by immunoblotting that drebrin was expressed in various bladder cancer cells (Figure 1B) with a plasma membrane distribution in 5637 invasive urothelial cancer cells (arrows, Figure 1C). To further validate the interaction between progranulin and drebrin, we performed confocal microscopy analysis. We detected drebrin colocalization with progranulin in punctate vesicles (Figure 1D) suggesting that this interaction occurs upon progranulin internalization from cell membrane. The colocalization was further validated by line scanning confocal (bottom panel of Figure 1D), strongly supporting the notion that drebrin is a novel binding partner of progranulin.

**Figure 2:**
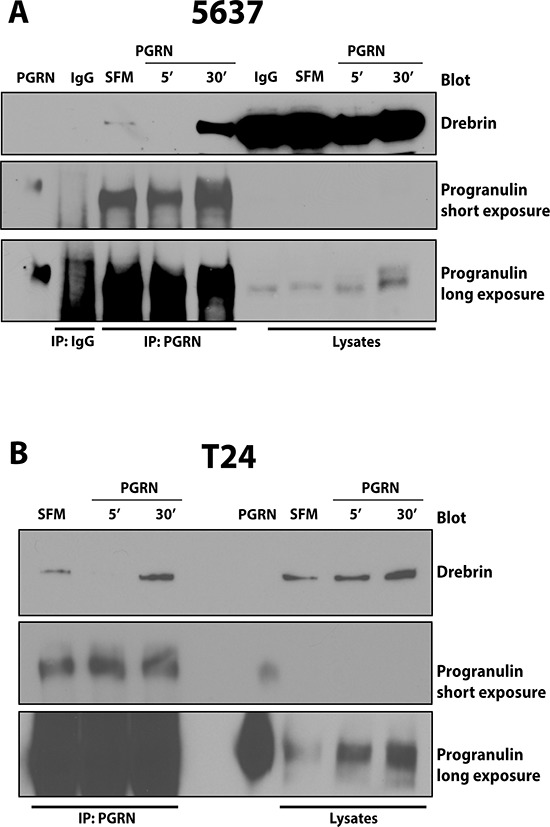
Progranulin coprecipitates with drebrin in 5637 and T24 urothelial cancer cells **(A)** Serum-starved (SFM) 5637 and **(B)** T24 cells were stimulated with recombinant progranulin (80 nM) for 5 and 30 min (5′ and 30′). progranulin interaction with drebrin was assesses by coimmmunoprecipitation experiments. 3 mg of lysates from 5637 (A) and T24 (B) cells were immunoprecipitated with anti-progranulin polyclonal antibodies. Unrelated IgG (IgG) was used as control (A) Drebrin was detected by immunoblot using anti-drebrin monoclonal antibodies. Blots are representative of two independent experiments.

Next, we confirmed the interaction between progranulin and endogenous drebrin by co-immunoprecipitation assays in 5637 cells (Figure 2A). Drebrin was detectable in complex with endogenously expressed progranulin at low levels in unstimulated cells and this association decreased after 5 min of progranulin stimulation (Figure 2A, 5′). On the contrary, 30 min of progranulin stimulation strongly enhanced the progranulin/drebrin interaction (Figure 2A, 30′). Drebrin was not detectable when lysates of 5637 cells were immunoprecipitated with an unrelated antibody used as control (Figure 2A, IgG). These results were confirmed by repeating coimmunoprecipation experiments in T24 cells, where we detected the same kinetics of association between progranulin and endogenous drebrin (Figure 2B). Longer exposure of progranulin blots are included to show endogenous progranulin in total lysates (Figure 2A and 2B). To confirm the results obtained by anti-progranulin antibodies, we performed reciprocal co-IP and immunoprecipitated the complex using anti-drebrin antibodies. We tried two commercially available antibodies but both antibodies showed an extremely low ability to immunoprecipitate endogenous drebrin, thus preventing us in detecting progranulin in complex with drebrin. (Not shown)

Collectively, these results indicate that drebrin complexes with progranulin and may play a role in regulating progranulin-induced biological responses in bladder cancer cells.

### Drebrin regulates progranulin-induced biological responses in urothelial cancer cells

**Figure 3:**
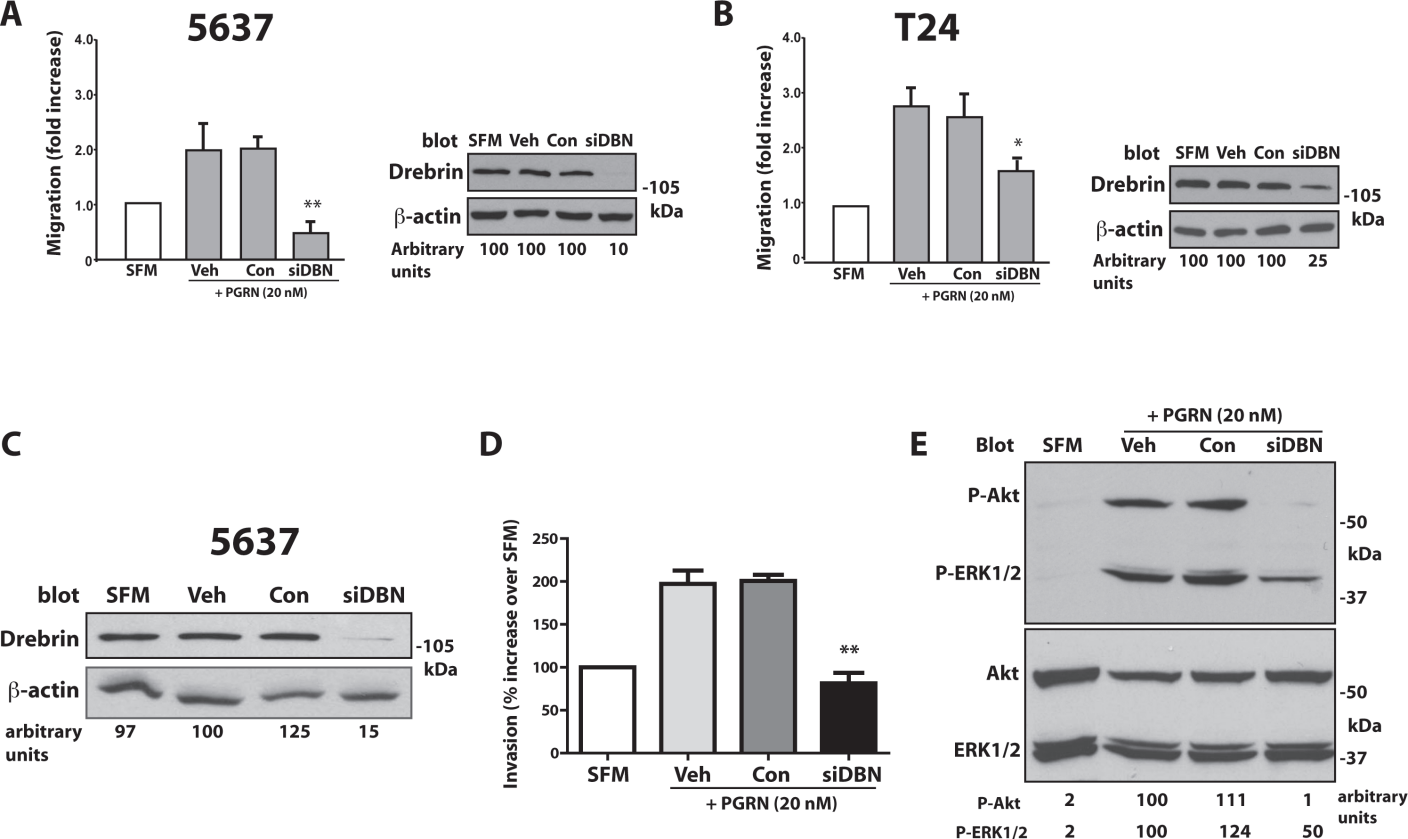
Drebrin modulates progranulin-induced motility and signaling of urothelial cancer cells **(A and B)** 5637 and T24 cells were transiently transfected with vehicle, control oligos or On-Target siGenome pool of drebrin-specific oligos. 48 h post-transfection cells were transferred to SFM or SFM supplemented with progranulin (40 nM) and counted for migration after 18 h. Data are the average of three independent experiments ±SD. *P < 0.05; **P < 0.01. Drebrin levels were assessed by immunoblotting with anti-drebrin polyclonal antibodies. Densitometric analysis is expressed as arbitrary units. **(C)** Drebrin-depleted 5637 cells were assessed for invasive ability through Matrigel **(D)** as described in Materials and Methods. Data are the average of three independent experiments ±SD. **P < 0.01. **(E)** Activation of Akt and ERK1/2 in drebrin-depleted 5637 cells was determined by immunoblotting using phospho-specific antibodies. Densitometric analysis is expressed in arbitrary units. Blot is representative of three independent experiments.

We have previously established the critical role of progranulin in promoting motility of bladder cancer cells [10, 11]. Thus, as an initial step to determine drebrin function in progranulin-mediated responses in urothelial cancer cells, we transiently depleted endogenous drebrin in 5637 and T24 cells by siRNA approaches and assessed cell migration. We achieved about ~90 and 75% depletion of endogenous drebrin in 5637 (Figure 3A) and T24 (Figure 3B) cells, respectively as compared to cells treated with either vehicle or scrambled siRNA. Suppression of drebrin expression led to a robust inhibition of migration in both cell lines in response to progranulin stimulation (Figure 3A and 3B).

As drebrin is important for progranulin-evoked cell migration, we hypothesized that drebrin may also regulate the ability of urothelial cancer cells to invade through a 3D extracellular matrix upon progranulin stimulation. Thus, we used Matrigel-coated filters to examine invasive ability of 5637 cells depleted of endogenous drebrin (Figure 3C). Following exposure to progranulin (40 nM), there was a marked increase in the ability of vehicle- and control oligo-transfected 5637 cells to invade a 3D matrix (Figure 3D) while drebrin knock-down significantly (**p < 0.01) reduced the invasive capacity of these cells (Figure 3D).

As progranulin-induced motility and invasion requires the activation of Akt and MAPK pathways [9–11], we sought to determine whether drebrin may regulate progranulin-dependent signaling and assessed by immunoblotting Akt and ERK1/2 activation in drebrin-depleted 5637 cells (Figure 3E). Drebrin knockdown almost completely abolished progranulin-induced Akt activation, and caused > 50% reduction in ERK1/2 phosphorylation as compared to vehicle or siRNA control-transfected 5637 cells (Figure 3E). The negative effect of drebrin depletion on progranulin signaling was reproducible in T24 cells but it more severely affected ERK1/2 activation as compared to Akt signaling (data not shown).

These results indicate that drebrin is a critical protein component for progranulin-mediated activation of Akt/MAPK pathways leading to cell migration and invasion and may indeed function as an essential component of a progranulin signaling complex in bladder cancer cells.

### Drebrin modulates progranulin-induced actin cytoskeleton remodeling

**Figure 4:**
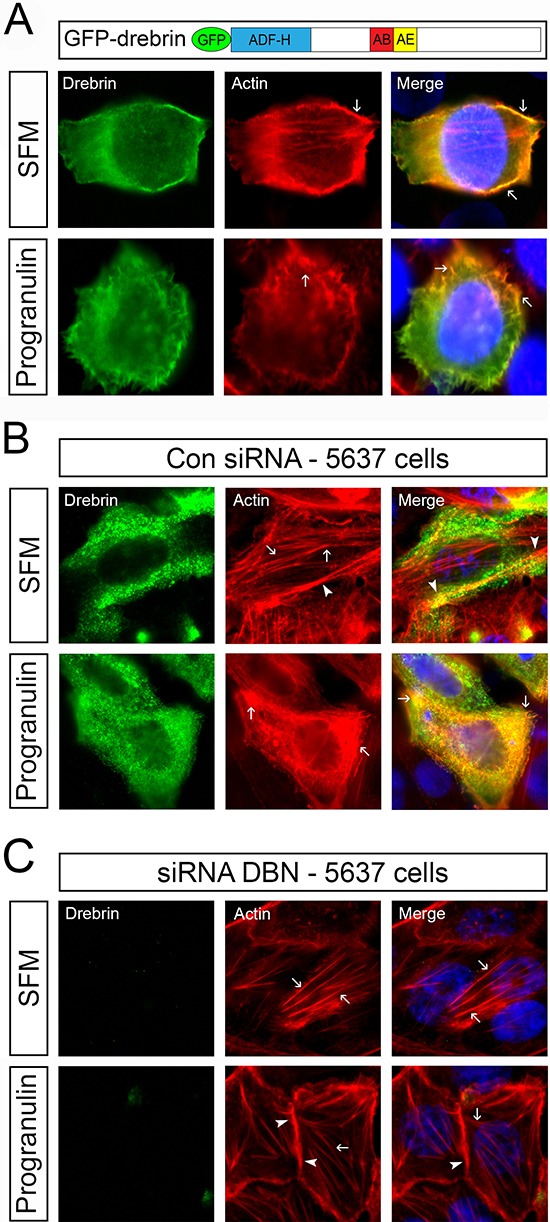
Drebrin is critical for progranulin-mediated F-actin remodeling **(A)** GFP-tagged wild type drebrin construct has been previously described [23]. (ADF-H) actin-depolymerizing factor homology domain. AB: actin binding domain. AE: adult specific exon. 5637 cells were transiently transfected with the GFP-tagged drebrin construct, serum-starved for 24 h and then stimulated with progranulin (40 nM) for 30 min. F-actin structures were assesses by immunofluorescence analysis as described in Material and Methods using Rhodamine-phalloidin. Pictures are representative of at least 10 independent fields from three independent experiments. An average of 300 cells was examined for each condition. Arrows indicate various F-actin structures. 5637 cells were transiently transfected with control oligos **(B)** or On-Target siGenome pool of drebrin-specific oligos (Dharmacon) **(C)** serum-starved for 24 h and stimulated with progranulin (40 nM) for 30 min. Immunofluorescence analysis was performed as described in Material and Methods. F-actin was detected using anti-phalloidin staining. Drebrin levels were analyzed using anti-drebrin monoclonal antibodies. Pictures are representative of at least 10 independent fields from three independent experiments. An average of 300 cells was examined for each condition. Arrows indicate F-actin networks while arrowheads identify cortical actin.

Cancer cell motility and invasion require a change in cellular morphology associated with actin remodeling [21, 22]. Because drebrin binds F-actin [19, 20], we investigated whether progranulin stimulation of 5637 cells would affect the ability of drebrin to bind F-actin and mediate actin remodeling. To this end, we transiently transfected a GFP-tagged drebrin protein and assessed by immunofluorescence analysis the resultant F-actin network, visualized by rhodamine-phalloidin labeling. In serum-starved 5637 cells, full length GFP-drebrin (residue 1–707) staining was diffuse in the cytoplasm and colocalized with cortical F-actin (Figure 4A, arrows). Interestingly, progranulin stimulation induced redistribution of drebrin in F-actin-enriched spikes [23] at the membrane edge of 5637 cells (Figure 4A, arrows).

To further confirm the role of drebrin in regulating progranulin-induced F-actin remodeling, we depleted 5637 cells of endogenous drebrin and analyzed F-actin network by rhodamine-phalloidin staining. Unstimulated siRNA control-transfected 5637 cells showed a well-organized F-actin network (arrows) and cortical actin (arrowhead) (Figure 4B, SFM), which was severely compromised after progranulin stimulation (Figure 4B, Progranulin). Significantly, drebrin-depleted 5637 cells showed no difference in F-actin remodeling between unstimulated and progranulin-stimulated 5637 cells (Figure 4C), which maintained the organized F-actin network (arrows) and intact cortical actin (arrow heads) after progranulin stimulation (Figure 4B).

Collectively, these results clearly suggest that drebrin regulates progranulin-induced cell motility of bladder cancer cells by modulating progranulin-mediated F-actin remodeling.

### Drebrin modulates anchorage-independent growth and *in vivo* tumor formation

**Figure 5:**
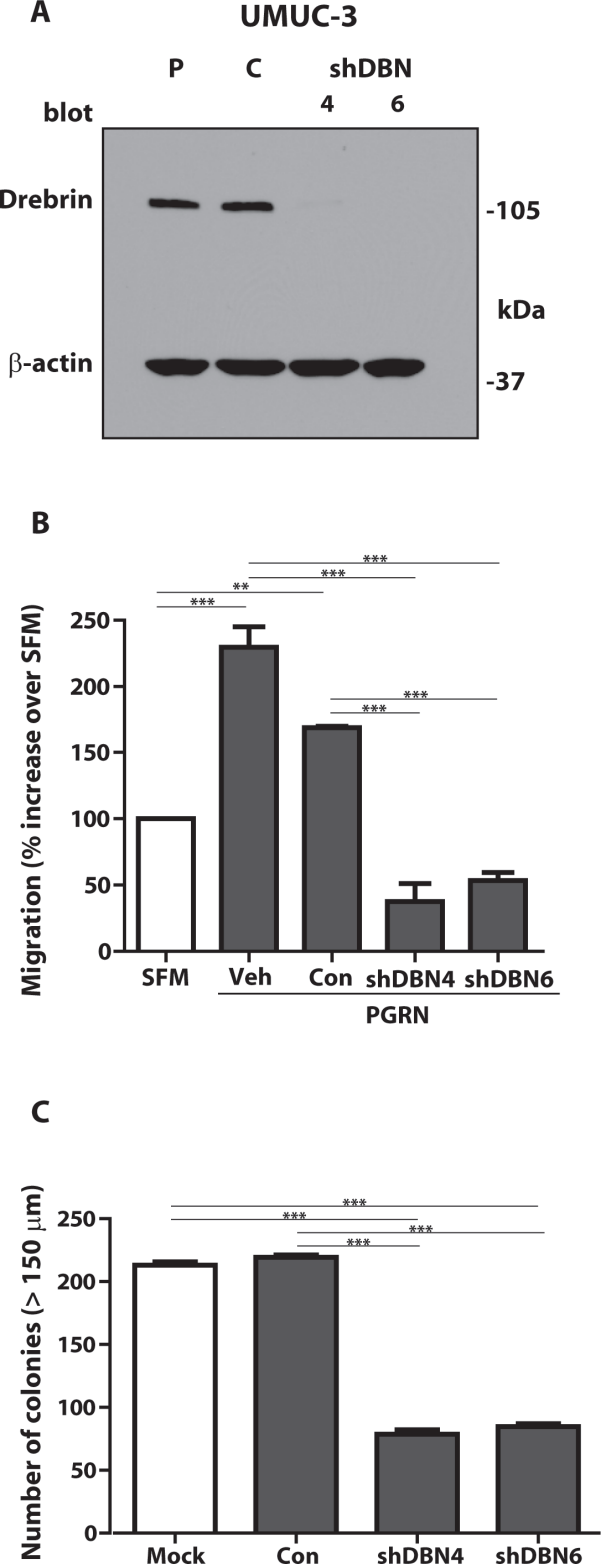
Stable depletion of endogenous drebrin in tumorigenic UMUC-3 urothelial cancer cells inhibits motility and anchorage-independent growth **(A)** The generation of UMUC-3/pRS-control and UMUC-3/pRS-shDBN cells has been described in Materials and Methods. Drebrin expression in lysates from parental (P), control- (C) or shRNA drebrin-transfected (shDBN) UMUC-3 cells was detected by immunoblot. **(B)** Migration of the various UMUC-3 cell lines was performed using transwells as described in detail in Materials and Methods. The experiment is the average of three independent experiments run in duplicates ±SD. **P < 0.01. ***P < 0.001. **(C)** Anchorage-independent growth was measured by colony formation in soft-agar as previously described [10]. Colonies > 150 μM were counted. The experiment is the average of three independent experiments run in duplicates ±SD. ***P < 0.001.

To ascertain drebrin activity in bladder cancer progression, we transfected a drebrin-specific shRNA-expressing plasmid in UMUC-3 urothelial carcinoma-derived cells. We choose these malignant cells as UMUC-3 cells form colonies in soft-agar and are tumorigenic in mice [24–27]. After selection in puromycin-containing media, we isolated two pools (mass cultures) of UMUC-3 cells where drebrin expression was abolished (Figure 5A, shDBN 4 and 6) as compared to either parental (P) or control-transfected (C) cells (Figure 5A). Notably, the drebrin-depleted UMUC-3 (UMUC-3^shDBN^) cells showed a markedly-attenuated migratory ability in response to progranulin stimulation (***p < 0.001, Figure 5B). More importantly, UMUC-3^shDBN^ cells were significantly impaired in forming colonies in a soft-agar assay (***p < 0.001, Figure 5C), indicating that drebrin exerts an important role in regulating anchorage-independent growth of bladder cancer cells.

**Figure 6:**
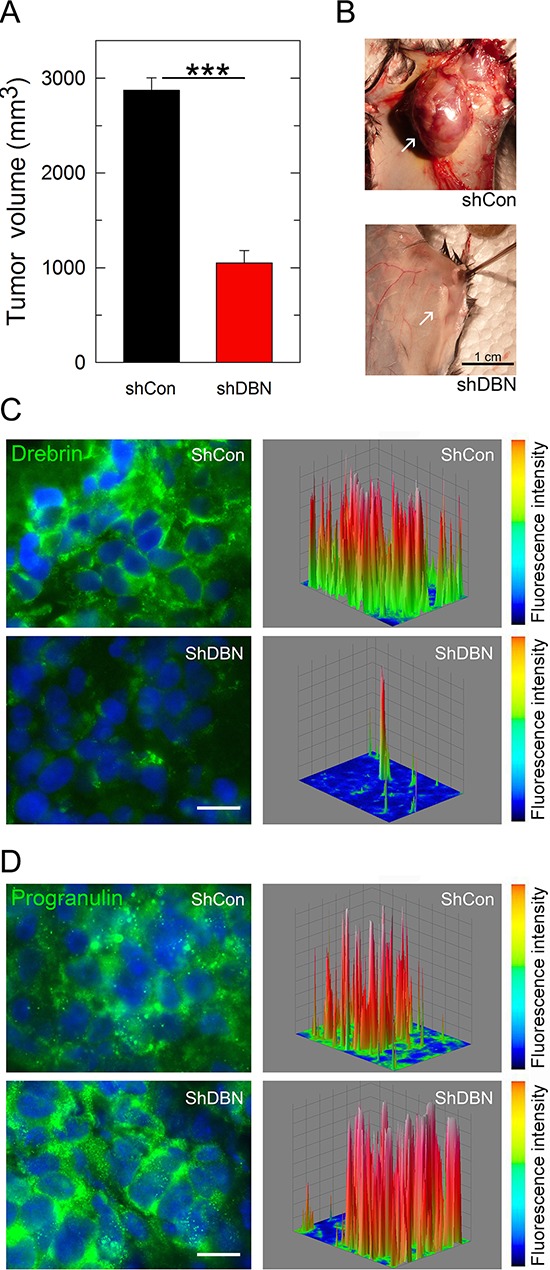
Drebrin regulates tumor formation *in vivo* **(A)** Tumor growth plot of mice (*n* = 18) injected with UMUC-3/sh-control (shCon) and UMUC-3/sh-drebrin (shDBN) cells at day 52. ***P < 0.001. **(B)** Representative macroscopic photographs of UMUC-3 control (shCon) and drebrin-depleted (shDBN) tumors at day 52. **(C and D)** Tumors extracted from UMUC-3/sh-control (shCon) and UMUC-3/sh-drebrin-injected (shDBN) mice were analyzed for drebrin (C) and progranulin (D) expression by immunofluorescence analysis. Three-dimensional surface plots are indicative of drebrin (C) and progranulin (D) expression levels in the relative fluorescence images. Accompanying scale bars on the right depict signal intensity.

Based on this finding that drebrin regulates anchorage-independent growth of UMUC-3 bladder cancer cells, we generated mouse xenograft models and investigated whether targeting drebrin could suppress the ability of UMUC-3 cells to form tumors *in vivo*. UMUC-3 shScr (UMUC-3^shScr^) control and UMUC-3^shDBN^ cells were implanted subcutaneously into the left and right flanks respectively of 6 week-old *Rag2*–/– mice which lack in the ability to initiate V(D)J rearrangement, leading to a severe immunocompromised phenotype. Once tumors were established, tumor sizes were monitored until the largest tumor reached ~2000 mm^3^ in size. Importantly, All control UMUC-3^shScr^ cells generated tumor xenografts in contrast to the debrin-depleted UMUC-3^shDBN^ cells which generated tumors only in ~30%, and in addition, the average tumor volume was significantly smaller than control (***p < 0.001, Figure 6A and 6B). To confirm that the tumor xenografts had indeed reduced expression of drebrin, we performed immunofluorescence analysis on frozen sections of tumors after the animals were sacrificed. Indeed, we found that drebrin levels were significantly depleted in the UMUC-3^shDBN^ xenografts (Figure 6C). On the contrary, drebrin depletion did not affect progranulin levels, which were very similar in control and drebrin-depleted UMUC-3 cells (Figure 6D).

Collectively, these findings provide a strong evidence for a role for drebrin in regulating tumor formation *in vivo* and suggest a drebrin mechanism of action which is independent of a possible effect on progranulin levels. Because we have preliminary evidences that progranulin depletion affects as well tumor formation *in vivo*, these results additionally support the hypothesis that drebrin functions by regulating downstream progranulin signaling.

### Drebrin expression is upregulated in bladder cancer tissues

**Figure 7:**
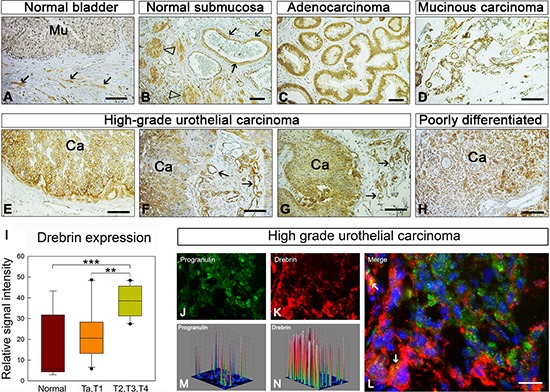
Drebrin is upregulated in bladder cancer tissues **(A–H)**, Panels of light micrographs depicting the distribution of immunoreactive drebrin in normal bladder (A and B) and various types of urinary cancers as indicated (C–H) Notice that in normal bladder, drebrin is essentially not expressed in the mucosa (Mu, a) but is expressed in the submucosal blood vessels and nerves (arrows and arrowheads, respectively, panel B). Drebrin is expressed at high levels in the epithelial components of all cancers (Ca) studied and often in the tumor vasculature (arrows in panels f and g). Bars = 100 μm. **(I)** Quantification of the drebrin staining was done using ImageJ software. Various images of normal (*n* = 9); Ta, T1 (*n* = 10) and T2, T3, T4 (*n* = 13) urothelial carcinoma tissues were analyzed. Briefly, the threshold of each image was adjusted in order to show only the specific staining. The representative areas of staining were then quantified and plot using SigmaPlot. **(L–J)** Drebrin and progranulin expression in frozen high grade urothelial carcinoma tissues was analyzed by immunofluorescence analysis. Arrows (L) indicate some areas of colocalization. Three-dimensional surface plots are indicative of progranulin **(M)** and drebrin **(N)** expression levels in the relative fluorescence images.

Next, we assessed whether drebrin expression is altered in human bladder cancer. To this end, we utilized a human bladder tumor microarray containing 45 validated cases in duplicate of various types of bladder cancers and normal bladder tissue (AccuMax™ array). In normal bladder tissue, drebrin was only detectable primarily in the submucosal mesenchyme (arrows, Figure 7A). Higher magnification view showed that drebrin was primarily expressed by vascular smooth muscle cells (arrows, Figure 7B) and nerves (arrowheads, Figure 7B). Notably, in all malignant tissues examined there was a marked upregulation of drebrin. Drebrin was markedly elevated in the epithelial components of both highly and poorly differentiated urothelial cancer (Figures 7C–7H). In addition, the angiogenic component of various bladder cancers, evident in the high grade transitional cell carcinomas (arrows, Figures 7F and 7G), showed enhanced expression of drebrin, suggesting that it might be also involved in positively regulating tumor angiogenesis. Significantly, the staining intensity index on drebrin expression levels of urothelial carcinoma tissues demonstrated that drebrin expression in high grade T2, T3 and T4 tumors is significantly increased over lower grade (Ta, T1) (**p < 0.01) and normal bladder tissues (***p < 0.001) (Figure 7I). In addition progranulin and drebrin expression partially colocalized in high grade urothelial carcinoma tissues, as demonstrated by immunofluorescence analysis on a frozen high grade urothelial carcinoma tissue (Figure 7J–7L).

## DISCUSSION

We have discovered a novel interaction between the growth factor progranulin and the F-actin-binding protein drebrin and provide several lines of evidence that underscore a key role for drebrin in regulating progranulin bioactivity in bladder cancer. First, we show that drebrin is expressed in urothelial carcinoma-derived cell lines where it associates with progranulin as demonstrated by pull-down experiments, proteomic approaches, coimmunoprecipitation assays and colocalization experiments using confocal laser microscopy. Second, we show for the first time that drebrin is required for progranulin-induced motility, invasion and activation of the Akt/MAPK pathways in two malignant urothelial cells. Third, we provide robust evidence that drebrin regulates progranulin-dependent F-actin remodeling and that this bioactivity also requires drebrin expression. Fourth, we show that tumorigenic UMUC-3 cells stably depleted in drebrin form fewer and smaller tumors in immunocompromised animals than the corresponding wild-type cells. Finally, we show that drebrin expression in upregulated in several human bladders cancers. Both highly and poorly differentiated bladder cancers express high levels of drebrin in the tumor proper. Notably, drebrin expression in high grade T2, T3 and T4 tumors is significantly increased over lower grade (Ta, T1) and normal bladder tissues.

Debrin was originally identified in the brain of chick embryos [20, 28] and it is expressed in two isoforms: an embryonic type (drebrin E) and an adult type (drebrin A), whose sequence is almost identical except for an internal insert sequence in drebrin A, but no clear functional differences have be so far described [20]. Most of the past work on drebrin expression and activity has been focused on neurons, in which drebrin binds F-actin and controls actin filament dynamics, neurite morphology and outgrowth [23, 29]. By affecting actin remodeling, drebrin plays a significant role in regulating cell motility in the physiology [30–32] and pathology of neuronal cells [33]. Drebrin expression has been more recently detected in non-neuronal tissues and cells, including stomach and kidney epithelia [34] and T cells, where drebrin binds the chemokine receptor CXCR4 and regulates immune synapsis [35].

Our current findings provide the first evidence of drebrin expression and interaction with the growth factor progranulin in urothelial-carcinoma derived cells where drebrin exerts an essential role in modulating progranulin-induced biological responses.

The progranulin-interactive drebrin fragment we isolated by mass spectrometry is conserved between drebrin A and E isoforms, and the anti-drebrin antibodies used in these studies do not discriminate between the different drebrin isoforms. However, because drebrin A is considered the neuron-specific isoform, while drebrin E expression is more ubiquitous [36], we can reasonably assume that bladder cancer cell lines likely express drebrin E. We have transiently expressed both drebrin isoforms in 5637 cells and we could not detect any differences in their ability to regulate progranulin-dependent F-actin remodeling.

Our previous work has established an essential role for progranulin in modulating migration and invasion of bladder cancer cells. Progranulin acts as an autocrine growth factor and regulates motility in part by activating paxillin and promoting paxillin colocalization with FAK at dynamic focal adhesion sites at the leading edge of migrating cells [10, 11]. Notably, progranulin stimulation of 5637 urothelial cancer cells does not significantly enhance cell proliferation [9] indicating that the ability of progranulin to promote cell motility is dissociated from a potential effect on cell growth.

Progranulin stimulation of 5637 cells induces drebrin redistribution at previously described F-actin spikes [23], which are morphologically very similar to dynamic adhesions at the membrane edge of motile 5637 cells [10, 11]. Thus, we propose that drebrin, by regulating F-actin remodeling, would modulate progranulin-dependent focal adhesion turnover of migrating bladder cancer cells. However, whether drebrin functionally interacts with paxillin and/or FAK at focal adhesions remains to be established. Significantly, recent experiments from our laboratories have demonstrated that the FAK homolog Proline-Rich Tyrosine Kinase 2 (Pyk2) is upregulated in bladder cancer tissues compared to normal tissue controls and plays a more important role than FAK in regulating IGF-I-induced motility and invasion of urothelial carcinoma cells [37]. We have preliminary evidence supporting a role of Pyk2 in progranulin-dependent signaling of urothelial cancer cells; however, it remains to be established whether Pyk2 would interact with drebrin and contribute with drebrin to the regulation of F-actin remodeling and/or focal adhesion turnover.

A limitation of our studies is that, although we have demonstrated by several independent approaches that progranulin interacts with drebrin, we cannot totally exclude the possibility that this interaction may not be direct but mediated through the formation of a multiprotein complex, which may regulate early events of progranulin signaling. As drebrin is not a trans-membrane protein, the interaction with progranulin is likely occurring at early stages of progranulin internalization from the cell membrane. However, the mechanisms regulating progranulin uptake and endocytosis are very poorly characterized, especially in bladder cancer cells. As we mentioned in the Introduction, in neuronal cells progranulin interacts with sortilin, which promotes progranulin internalization and targeting for lysosomal degradation [14]. We have discovered that sortilin is expressed in urothelial cancer cell lines suggesting that sortilin might complex with progranulin and drebrin in early endocytic compartments and may play any role in modulating early events of progranulin signaling.

Our studies point out to a very important role for drebrin in regulating bladder cancer initiation and progression. Drebrin is essential for progranulin-induced signaling of urothelial cancer cells as in fact drebrin depletion significantly inhibits the activation of both the Akt and MAPK pathways, which are essential for progranulin-dependent motility and invasion of urothelial cancer cells [10, 11]. More importantly, drebrin depletion profoundly affects the ability of urothelial cancer cells to grow in anchorage-independency and form tumors in xenograft models suggesting the possibility that drebrin may not only affect progranulin-dependent responses but also regulate additional pathways, which may converge with progranulin signaling thereby promoting bladder tumor initiation and possibly progression.

There is a great interest at the moment in the identification of novel biomarkers for prognosis and treatment selection of advanced bladder cancers [38–40]. Our results demonstrated drebrin overexpression in various bladder cancer tissues and drebrin levels are enhanced in high grade urothelial carcinoma compared to lower grade and normal urothelial tissue controls. In addition drebrin partially colocalized with progranulin in high grade urothelial carcinoma tissues. These results suggest that drebrin expression in bladder cancer tissue may work in conjunction with progranulin levels as a novel biomarker for bladder cancer and may identify tumors likely to progress to the invasive phenotype. However, additional studies including the analysis of metastatic bladder cancer tissue samples are required to clearly establish a role for drebrin in tumor progression.

In summary, our studies have identified the F-actin protein drebrin as a novel progranulin interacting protein critical for motility, invasion, anchorage-independent growth and tumor formation *in vivo* of urothelial cancer cells. Drebrin may constitute therefore a novel target for therapeutic intervention in bladder tumors.

## MATERIALS AND METHODS

### Cell lines

Urothelial carcinoma-derived human 5637, T24 and UMUC-3 cells were obtained by ATCC (Manassas, VA, USA).

5637 and T24 cells were maintained in RPMI medium with 10% fetal bovine serum (FBS). UMUC-3 cells were maintained in MEM with EARL medium with 10% FBS. Serum-free medium (SFM) is DMEM supplemented with 0.1% bovine serum albumin and 50 μg/ml of transferrin (Sigma-Aldrich, St Louis, MO, USA).

### Pull-down assays with recombinant progranulin and proteomic analysis

Serum-starved 5637 cells were stimulated with progranulin (40 nM) for 5 min and lysates collected in NP-40 lysis buffer containing Protease and Phosphatase Inhibitor Cocktail (Thermo Scientifics, Waltham, MA USA). After preclearing for 1 h with Ni-NTA-agarose beads (QIAGEN, Valencia, CA, USA), lysates (10 mg) were first incubated with or without 7 μg of recombinant HIS-tagged progranulin overnight a 4°C and then with Ni-NTA-agarose beads for 1 h. Beads were washed with lysis buffer and resuspended in Laemmli buffer. Samples were separated by SDS-PAGE, visualized with Coomassie blue staining and processed for mass spectrometry at the Proteomic Core Facility of the Kimmel Cancer Center. Bands were excised and digested with trypsin after reduction and alkylation. Peptides were separated on a 10-cm C18 column and analyzed on a Thermo LCQ 3D ion trap using the nano-source. MS/MS spectra were searched on a local Mascot server against the Swissprot database. All peptides were identified with a confidence of at least 95%.

### Confocal microscopy

5637 cells on slides were serum-starved overnight and treated with progranulin (40 nM) for 5 min. Cells were then washed with PBS and fixed with 4% PFA for 30 min at room temperature. Subsequently, slides were subjected to immunofluorescence and confocal analysis as previously described [9, 10, 37, 41–43]. Primary antibodies were anti-progranulin polyclonal (USBiologicals, Swampscott, MA, USA) and anti-drebrin monoclonal antibodies (Abcam, Cambridge, MA, USA). Secondary antibodies were goat anti-mouse IgG Alexa Fluor^®^ 488 and goat anti-rabbit IgG Alexa Fluor^®^ 594 antibodies (Invitrogen, Grand Island, NY, USA). Confocal analysis was carried out using a 63x, 1.3 oil-immersion objective of a Zeiss LSM-78 confocal laser-scanning microscope. To determine colocalization of the two proteins, Z-stack series were acquired maintaining the same slice interval. All images were analyzed using ImageJ and Adobe Photoshop CS6 (Adobe Systems). Line scanning plots were performed utilizing SigmaPlot software.

### Co-immunoprecipitation and immunoblotting

Serum-starved 5637 and T24 cells were stimulated for 5 and 30 min with 80 nM progranulin and lysates (3 mg) immunoprecipitated with anti-progranulin polyclonal antibodies (USBiologicals). Progranulin was detected by immunoblot using anti-progranulin polyclonal antibodies (USBiologicals) while drebrin expression was detected using either anti-drebrin polyclonal antibodies (Sigma-Aldrich) or anti-drebrin monoclonal antibodies (Abcam).

### Drebrin depletion by siRNA approaches

Transient depletion of endogenous drebrin in 5637 and T24 cells was achieved by siRNA approaches using vehicle, control oligos or On-Target siGenome pool of drebrin-specific oligos (Dharmacon, Lafayette, CO, USA). Cells were transfected with 400 pM of oligos using the TransIT-TKO^®^ Transfection Reagent (Mirus, Madison, WI, USA). Biological responses and drebrin expression levels were assessed after 72 h post-transfection.

### Migration, wound healing and invasion assays

HTS FluoroBloks™ inserts (Becton Dickinson, Durham, NC, USA) were saturated with PBS-1% bovine serum albumin for 2 h at room temperature. Serum-starved cells were labeled with DiI (Molecular Probes, Grand Island, NY, USA) for 20 min at 37°C and seeded in the HTS FluoroBloks™ upper chamber in SFM or SFM supplemented with progranulin (40 nM) and incubated at 37°C for 18 h. Membranes were fixed in 4% paraformaldehyde, mounted on slides and migrated cells were counted and photographed with a Zeiss Axiovert 200 M cell live microscope. Cell invasion was assessed by using BD Matrigel™ Invasion Chambers (BD Biocoat, Bedford, MA, USA) with 8.0 μm filter membranes. Cells (5 × 10^4^) in 200 μl of SFM were plated onto each filter, and 750 μl of SFM or SFM supplemented with progranulin (40 nM) in the lower chamber. After 24 h filters were washed, fixed, and stained with Coomassie Brilliant Blue. Cells on the upper surface of the filters were removed with cotton swabs. Cells that had invaded to the lower surface of the filter were counted under the microscope.

### Immunoblot detection of activated signaling pathways

Serum-starved cells were then stimulated with progranulin (40 nM) for 10 min. The activation of Akt and ERK1/2 was analyzed by western immunoblot using phospho-specific antibodies (Cell Signaling Technology, Beverly, MA, USA) as described [44]. Total levels of Akt and ERKs were monitored with anti-Akt and anti-ERK1/2 antibodies (Cell Signaling Technology).

### Drebrin expression and F-actin remodeling

The GFP-drebrin fusion proteins GFP-drebrin A wt was previously described [23]. 5637 cells were transfected on slides using the TransIT^®^-Prostate Transfection Kit (Mirus). 48 h after transfection, cells were serum-starved for 24 h and then treated with either 1X PBS or 40 nM of progranulin for 30 min. Cells were fixed for 30 min at room temperature with 4% paraformaldehyde in 1X PBS and permeabilized for 30 seconds with 0.01% Tween in 1X PBS. F-actin staining was assessed by Rhodamine-phalloidin (Invitrogen) for 20 min and then fixed with Vectashield (Vector, Burlingame, CA, USA). Drebrin expression was detected using an anti-drebrin polyclonal antibody (Sigma-Aldrich) followed by a goat anti-rabbit IgG Alexa-Fluor 594 secondary antibody. Immunofluorescence analysis was performed using a LEICA DM5500B fluorescent microscope.

### Generation of progranulin-depleted UMUC-3 cells

UMUC-3 cells stably depleted of endogenous drebrin were generated by transfecting the pRS vector, the pRS-shRNA-control (scrambled shRNA) and pRS/shDBN plasmids (OriGene Technologies, Inc., Rockville, MD, USA) using the TransIT^®^-Prostate Transfection Kit (Mirus). Cells were selected in medium supplemented with 2 μg/ml of Puromycin. After selection, pools of drebrin-depleted UMUC-3 cells were tested for drebrin expression levels by immunoblot using anti-drebrin monoclonal antibodies (Abcam).

### Colony formation assay in soft-agar

Colony formation in soft-agar was performed as previously described [10]. Cells were counted after four weeks in culture. Colonies > 150 μm were scored as positive.

### Tumor xenografts

Experiments using 7- to 10-week-old *Rag*2–/– mice were carried out according to protocols approved by the Institutional Review Board of Thomas Jefferson University. 4 × 10^6^ UMUC-3/sh-control and sh-drebrin cells were injected subcutaneously in two distinct sites of mice flanks (control cells in the upper-left flank and drebrin-depleted cells in the lower-right flank). Three independent experiments with 6 mice each were performed (*n* = 18). Once tumors were established, tumor growth was measured every 2 days with a micro-caliper utilizing the following formula: *V* = a(b^2^/2), where a and b represent the larger and small diameters, respectively. When tumors reached 2000 mm^3^ in size, mice were sacrificed and tumors surgically dissected and divided. One half was snap frozen in liquid nitrogen for further biochemical analysis, whereas the other half was embedded in OCT medium (Sakura Finetek, Torrance, CA, USA) and frozen at –20°C. 10 μm thick cryostat sections were cut from the blocks, mounted on slides and subjected to immunofluorescence analysis utilizing a mouse monoclonal anti-drebrin antibody (Santa Cruz, Dallas, Texas, USA).

Colocalization of progranulin and drebrin was assessed by immunofluorescence analysis on an unidentified high grade human bladder tissue using anti-drebrin and progranulin antibodies described above. Three-dimensional surface plots were created with ImageJ and represent drebrin and/or progranulin expression based on the intensity of the immunofluorescence signal.

### Immunohistochemical detection of drebrin in bladder cancer tissues

Drebrin expression was analyzed by immunohistochemistry on an AccuMax™ bladder cancer tissue microarray (Petagen Inc., Seodaemun-gu, Seoul, Korea) at the Translational Core Facility of the Kimmel Cancer Center. The anti-drebrin monoclonal antibody was used at a 1:200 dilution. Detailed specifications of the bladder tissue array can be found at: tissuearray.petagen.com/main/products.php?no=110.

Quantification of the drebrin staining in urothelial carcinoma tissues was done using ImageJ software. Various images of normal (*n* = 9); Ta, T1 (*n* = 10) and T2, T3, T4 (*n* = 13) urothelial carcinoma tissues were analyzed. Briefly, the threshold of each image was adjusted in order to show only the specific staining. The representative areas of staining were then quantified and plot using SigmaPlot.

### Statistical analysis

Results of multiple experiments are expressed as mean ± SD. All statistical analyses were carried out with SigmaStat for Windows version 3.10 (Systat Software, Inc., Port Richmond, CA). Results were compared using the two-sided Student's *t* test. Differences were considered statistically significant at *p* < 0.05.
